# Functional Variant rs4442975 Modulating FOXA1 Binding Affinity Can Influence Bone Marrow Suppression during Neoadjuvant Chemotherapy for Luminal A Type Breast Cancer

**DOI:** 10.1155/2019/7073498

**Published:** 2019-02-06

**Authors:** Xin Wang, Zhongxin Feng, Juan Li, Yanyan Han, Liyu Su, Feng Wang, Yan Yang, Yi Zhang

**Affiliations:** ^1^Department of Orthopaedic Surgery, Affiliated Hospital of Zunyi Medical University, Zunyi, 563000 Guizhou, China; ^2^Department of Hygiene Toxicology, School of Public Health, Zunyi Medical University, Zunyi, 563000 Guizhou, China; ^3^Department of Hematology, Affiliated Hospital of Zunyi Medical University, Zunyi, 563000 Guizhou, China; ^4^Department of Anesthesiology, Affiliated Hospital of Zunyi Medical University, Zunyi, 563000 Guizhou, China; ^5^Department of Endocrinology, Affiliated Hospital of Zunyi Medical University, Zunyi, 563000 Guizhou, China

## Abstract

The expression of the transcription factor FOXA1 is associated with the prognosis of estrogen receptor (ER)-positive breast cancer, and the genetic variant rs4442975 can affect FOXA1 function. Therefore, we investigated the association between rs4442975 and the efficacy of neoadjuvant chemotherapy for luminal A type breast cancer and evaluated its toxic side effects in a Chinese population. One hundred seventy-five patients with luminal A type breast cancer receiving neoadjuvant chemotherapy with a combination protocol of epirubicin and docetaxel (ET protocol) were enrolled in the study. Genotyping was performed in a randomized manner to identify candidate genetic variants. Unconditional logistic regression analysis was used to analyze the association of the variant with the efficacy and side effects of neoadjuvant chemotherapy. The results did not reveal any positive association with the efficacy of neoadjuvant chemotherapy, with an odds ratio (OR) of 0.73 (95% confidence interval = 0.27–1.94) in the additive model. However, analysis of the toxic side effects of neoadjuvant chemotherapy showed that rs4442975 was associated with bone marrow suppression, with an OR of 0.38 (95% confidence interval = 0.17–0.73,* p* = 0.005) in the dominant model. In summary, the functional genetic variant rs4442975 was associated with bone marrow suppression during neoadjuvant chemotherapy for luminal A type breast cancer. These results may help establish reliable molecular markers for predicting the prognosis of personalized treatment for luminal A type breast cancer and thereby contribute to the development of appropriate therapies.

## 1. Introduction

Breast cancer (BC) is a common malignancy that seriously threatens human health [1, 2] and is the primary cause of cancer-related death among young women in China [3]. The highly heterogeneous nature of BC stems from its multiple origins, which is because of the differences in the molecular pathways leading to the disease. This is evident from the variations in biological behavior and prognoses among patients with BC at the same clinical stage with identical pathological type [4]. With advanced molecular phenotyping methods, BC is classified into five subtypes based on gene expression signature: luminal A, luminal B, HER2, basal-like, and normal breast-like [5]. Luminal A type BC mainly presents with positive estrogen receptor (ER), negative epidermal growth factor receptor 2 (EGFR2), and low Ki-67 expression, accounting for 45–70% of all BC types [6, 7]. The Shanghai Breast Cancer Survival Study (SBCSS), which included a large sample size, reported that luminal A type accounts for 48.6% of all BC types in China; however, this number might be an underestimation [8]. Regardless of the absolute number, luminal A type BC is clearly the most common type of BC, and its response to treatment might be representative of the overall therapeutic efficacy of anti-BC therapies.

Neoadjuvant chemotherapy (NAC) for BC, also known as preoperative or induction chemotherapy, refers to the systemic delivery of cytotoxic drugs for BC* in situ* and is performed prior to local therapies such as surgery and radiotherapy. NAC significantly increases the pathological complete response (pCR) and survival rates for patients with no residual tumor or with only carcinoma* in situ*. A study by the National Surgical Adjuvant Breast and Bowel Project (NSABBP) reported that patients with pCR after NAC display a significant increase in disease-free survival (DFS) and overall survival (OS) [9], indicating that patients with BC are likely to benefit from effective NAC. However, the complete response (CR) rates do not increase for patients not responding to NAC, despite continuous treatment using a protocol with no cross-drug resistance. Therefore, NAC is primarily aimed at maximally reducing tumor size and increasing the breast-conserving and cure rates in patients. In addition, since the patients in the NSABBP study did not receive chemotherapy before NAC, the efficacy of this treatment was not affected by previous therapies. Therefore, this treatment approach provides a unique advantage for evaluating chemotherapeutic sensitivity and in helping to assess drug efficacy.

The pioneer transcription factor FOXA1 can tightly bind to dense chromosomes, unfold nucleosomes in the binding area, recruit new transcription factors, and promote the binding of other transcription factors to chromosomes [10]. Therefore, as a typical transcription factor, FOXA1 can not only activate or inhibit downstream targets, but also act as a pioneer transcription factor to modulate the binding of other transcription factors with target genes to affect downstream target gene expression. Thus, it participates in many biological events that cause carcinoma, such as cell cycle control, cell proliferation, epithelial–mesenchymal transition, cell migration and infiltration, and hormone metabolism [11]. In ER-positive (ER^+^) BC cell lines, almost all combinations of ER*α* with target genes and the expression of the related target genes are dependent on FOXA1 [12]. Meanwhile, studies using certain cellular models indicate that in* FOXA1*-silenced ER^+^ BC cell lines, both the binding of ER*α* with target genes and the transcriptional activity of the target genes are inhibited at the whole-genome level [13]. Therefore,* FOXA1* is considered a minimum-feature gene in BC and is a pivotal genetic marker for predicting the prognosis and outcomes of BC [14]. As a pioneer transcription factor, changes in the binding affinity of FOXA1 can affect ER recruitment [15]. Furthermore, BC is a hormone-dependent cancer, and ER plays a pivotal role in its development and progression; different expression levels of ER and FOXA1 are significantly associated with BC prognosis [16–18].

Unlike other types of BC, luminal A type BC is not sensitive to NAC; however, some patients with luminal A type BC may exhibit partial response (PR) [19, 20]. Therefore, an appropriate NAC program is essential to successfully treat this type of BC. To identify patients sensitive to chemotherapy and accordingly design personalized NAC for luminal A type BC, there is a need for reliable molecular signatures and biomarkers that can be used as predictive indicators of the efficacy of NAC. Since single effective biomarkers are lacking, Xu* et al*. [17] investigated the predictive effect of FOXA1 on the chemosensitivity of this BC subtype during NAC and reported that FOXA1 expression is significantly associated with the prognosis of ER^+^ BC.

A genome-wide association study (GWAS) [21] by Ghoussaini et al. demonstrated that the variant rs4442975, which disrupts the recruitment of FOXA1, was associated with BC risk in Europeans. Because the expression of FOXA1 is significantly associated with the prognosis of ER^+^ BC and the genetic variant rs4442975 can affect FOXA1 function [21, 22], the current study investigated the association between rs4442975 and the efficacy of NAC for luminal A type BC and its toxic side effects in a Chinese population. This will help identify reliable biomarkers to predict the efficacy of personalized therapies for luminal A type BC and to contribute to the development of appropriate therapies that will improve prognosis.

## 2. Materials and Methods

### 2.1. Study Subjects

Patients diagnosed with luminal A type BC and subjected to NAC with a combination protocol of epirubicin and docetaxel (ET protocol) between 2014 and 2016 at Affiliated Hospital of Zunyi Medical University were recruited as study subjects. The study subjects were unrelated, and all were Han women from Zunyi City (Guizhou Province) or from neighboring cities in the province. All the patients had ER^+^ BC. The inclusion criteria for patients with BC included histopathologically confirmed diagnosis, no history of carcinoma, and no previous local radiotherapy or chemotherapy prior to blood sample collection. Other specific inclusion criteria were as follows:

(1) Patients who underwent core-needle breast biopsy before NAC and presented with luminal A type BC that was ER^+^ and HER2^−^ and with Ki-67 < 15% based on immunohistochemistry analysis

(2) Patients who underwent NAC under the ET protocol for 4–6 weeks. In the first cycle of chemotherapy, the initial dose of either epirubicin or docetaxel was not less than 75 mg/m^2^

(3) Patients in whom cancer assessment was performed before chemotherapy or after every two cycles of chemotherapy with the assessment of therapeutic efficacy conforming with the Response Evaluation Criteria In Solid Tumors (RECIST) standards

(4) Patients who underwent radical surgery for BC (modified radical mastectomy or breast conservative surgery) after chemotherapy and then underwent a pathological assessment of therapeutic efficacy in accordance with Miller and Payne's (MP) grading system

There were no age or histological restrictions for the study group. The exclusion criteria for patients with BC included comorbid metastatic carcinoma, a history of another carcinoma, or the simultaneous incidence of two or more malignant tumors. The study was approved by the ethics committee of all participating institutions, and all study subjects provided written informed consent to participate in the study in accordance with the tenets of the Declaration of Helsinki.

### 2.2. Sample and Data Collection

Peripheral venous blood (5 ml) was collected from each of the participants before chemotherapy and used for DNA extraction. The general demographic data and clinicopathological data of the patients were obtained from medical records and personal interviews. The demographic data included age, marital and reproductive history, family history, smoking and drinking habits, and nationality. The clinicopathological data included staging, chemotherapy protocol, short-term outcome using the RECIST standard, the MP grading system to assess clinical and pathological therapeutic efficacies, and the NCI-CTC 3.0 standard to evaluate toxic responses. The data were entered for evaluation using Epidata 3.1 software. All data were entered in duplicate, followed by an inter-entry comparison to ensure data input accuracy. Participants who never smoked or smoked less than one cigarette a day for not more than one year were defined as “not smoking” and all other scenarios were considered as “smoking.” Drinking more than twice a week for at least one year was defined as “drinking” and other scenarios were considered as “not drinking.”

### 2.3. Genotyping

The genomic DNA was isolated from the blood samples collected from each participant using the RelaxGene Blood DNA System (Cat. #DP319-02; Tiangen, Beijing, China) according to the protocol of the manufacturer. The genetic variants were genotyped using a TaqMan single nucleotide polymorphism (SNP) genotyping assay and PCR using the 7900HT Fast Real-Time System (Applied Biosystems, Foster City, CA, USA). Genotyping was performed in a blinded and randomized manner. Approximately 5% of the random samples were genotyped twice, with the results being 100% concordant. All methods were performed in accordance with the approved guidelines.

### 2.4. Statistical Analyses

The odds ratios (ORs) and their 95% confidence intervals (CIs) were calculated using unconditional multivariate logistic regression analysis to estimate the association between rs4442975 and BC prognosis after adjusting for age, drinking, smoking, and menopausal status. All* P* values were based on two-sided analyses and* P* values < 0.05 were considered statistically significant. All statistical analyses were performed using SPSS version 18 software.

## 3. Results

### 3.1. Patient Demographic Characteristics

One hundred seventy-five patients with luminal A type BC were recruited for the study and underwent neoadjuvant chemotherapy using the ET protocol. The median age was 54 years (range, 33–75 years). Among the patients, 144 (82.29%) were menopausal. After NAC with the ET protocol, the PR rates were >80%; 112 patients (64.00%) presented with various degrees of bone marrow suppression; and 93 patients (53.14%) exhibited symptoms of gastrointestinal toxicity (Table 1).

### 3.2. Association of the Genetic Variant with the Efficacy of NAC

The genotypes of rs4442975 conformed to the Hardy–Weinberg equilibrium (*P* = 0.399). The minor allele frequency was 0.141, and the call rate was 95%. The association of rs4442975 with the therapeutic efficacy of NAC for luminal A type BC is shown in Table 2. There was no positive association between rs4442975 and the efficacy of NAC.

After chemotherapy, the patients were divided into two groups. One group presented with no therapeutic efficacy (stable disease; SD) or with deterioration of the disease (progressive disease; PD). The other group showed improved prognosis (pCR, CR, or PR). A comparison of the two groups after adjusting for factors including drinking, smoking, menopause, and age revealed that the OR of rs4442975 was 0.73 (95% CI = 0.27–1.94) in an additive model. Similar negative results were also observed in other models.

### 3.3. Association of the Genetic Variant with Toxic Side Effects of NAC

Toxic side effects including bone marrow suppression and gastrointestinal toxicity were observed after chemotherapy in the study participants. We analyzed the association between the candidate variant and the toxic side effects of the NAC for luminal A type BC. The association of rs4442975 with bone marrow suppression is shown in Table 3. Both the additive (OR = 0.39, 95% CI = 0.19–0.81,* P *= 0.011) and dominant models (OR = 0.36, 95% CI = 0.17–0.73,* P *= 0.005) indicated that mutation in rs4442975 was significantly associated with reduced risk of bone marrow suppression. The result of the additive model suggested that the risk of bone marrow suppression decreased by 62% with the increase in the occurrence of the mutant G allele. However, rs4442975 was not significantly associated with gastrointestinal toxicity (OR = 0.59, 95% CI = 0.30–1.41,* P *= 0.992 in the additive model).

## 4. Discussion

BC is the primary cause of cancer-related mortality among young women in China [23]. The identification of molecular features or biomarkers associated with BC prognosis therefore has important theoretical and application value. A combinatorial therapy including anthracyclines and taxanes is the standard protocol used as NAC for BC because this protocol demonstrates higher therapeutic efficacy than that of other protocols. However, this protocol elicits significant differences in individual responses. Currently, it is believed that genomic variations lead to individual differences in chemotherapeutic efficacies during the treatment of patients for tumors and other complex diseases, as patients bearing genetic variants might be sensitive to certain drugs. Single nuclear polymorphism is a common genetic variation that occurs in regulatory and coding regions of genes involved in important biological pathways, including those involved in cell proliferation, apoptosis, and DNA repair [24–26]. The polymorphisms may significantly affect gene expression or protein function and thereby the therapeutic efficacies of taxanes and anthracyclines. Therefore, identifying and analyzing functional genetic variations in genes associated with chemotherapeutic efficacies may help identify molecular markers or marker groups that may be used to predict chemotherapeutic efficacy.

FOXA1 functions as a pioneer transcription factor, which regulates transcription through the association of ER*α* with target genes and related target genes. ER*α* belongs to the nuclear receptor superfamily and promotes the association of cell proliferation transcription factors and multiple key target genes, thereby playing a crucial role in cancer cell proliferation and maintaining the development and growth of tumors. BC is a type of hormone-dependent cancer in which estrogen plays a pivotal role in its development and progression. The mechanism of action of estrogen includes ER*α* activation by its interaction with estrogen, which then regulates target gene expression by binding to the estrogen response element on the target gene. Since FOXA1 plays a pivotal regulatory role in ER*α*-mediated target gene expression, it is an important regulator of biological processes, including estrogen-mediated gene expression and BC cell proliferation. Furthermore, evidence suggests that FOXA1 is associated with drug reactions during chemotherapy [17, 27].

Single nuclear polymorphisms of rs4442975 are located near a putative regulatory element and are associated with allele-specific FOXA1 binding. It has been reported that the G-allele of this SNP reduces FOXA1 binding and hence results in reduced chromatin accessibility, cofactor recruitment, and long-range chromatin interactions [21, 22]. Furthermore, evidence suggests that this single nuclear polymorphism flanks a transcriptional enhancer that physically interacts with the promoter of insulin-like growth factor-binding protein 5 (IGFBP5), resulting in increased expression of this gene, which has known roles in BC biology [21]. This SNP has been reported to be associated with an increased risk of BC in European and American populations but not in the Chinese population [28]. In the current study, genetic variation of rs4442975 was associated with bone marrow suppression during NAC for luminal A type BC in a Chinese population. The additive model indicated that an increase in the occurrence of the mutant G allele might reduce the risk of bone marrow suppression.

Bone marrow stromal cells (BMSCs) synthesize and secrete insulin-like growth factor (IGF)-I and IGF binding protein (IGFBP). IGFBP5 has been suggested to be a key modulator in IGF-induced hematopoietic reactions [29]. Using whole-genome microarray to analyze gene transcription, Elsafadi et al. showed that IGFBP5 and interleukin-6 (IL-6) were upregulated in immortalized human BMSCs [30]. IL-6 signaling, which plays an important role in hematopoiesis and immunomodulatory function, is reported to be activated by the IGBP5 pathway [31]. Therefore, we speculated that the genetic variant rs4442975 might differentially affect myelosuppression by regulating the binding of transcription factor FOXA1 and the expression of the target gene* IGFBP5*. IL-6 exerts a vital effect on immunomodulation since the IGBP5 pathway interacts with IL-6 signaling. However, results from the current study indicated that rs4442975 was not associated with other toxic side effects (gastrointestinal toxicity) or chemotherapeutic outcomes.

The study had some limitations. Firstly, only age, smoking, alcohol use, and menopausal status were adjusted in the regression analysis, while other clinical or demographic factors might affect the assessment of variation risk. Secondly, unfortunately, we do not have any other independent patients, the results were obtained using a relatively small sample size; hence, the findings must be verified in the future using a larger sample size and other independent patients. Nevertheless, the present study provides a novel insight for future genome-wide association studies, as it is important to establish biomarkers associated with the efficacy and adverse effects of NAC for luminal A type BC.

## 5. Conclusions

In summary, we identified a locus positively associated with drug toxicity. The functional genetic variant rs4442975 was associated with bone marrow suppression during NAC for luminal A type BC. The results will be helpful in establishing reliable molecular markers and predicting the prognosis of personalized treatment for luminal A type BC, thereby contributing to the development of appropriate therapies.

## Figures and Tables

**Table 1:** Characteristics of the participants.

Variable	Number of cases (N = 175)
Median age (range)	54 (33–75)
Menopausal status	
Premenopausal	31 (17.71%)
Postmenopausal	144 (82.29%)
TNM stage	
TNM I-II	130 (74.29%)
TNM III-IV	45 (25.71%)
Efficacy of NAC	
Effective (pCR + CR + PR)	147 (84.00%)
Invalid (SD + PD)	28 (16.00%)
Bone marrow suppression	
Yes	112 (64.00%)
No	63 (36.00%)
Gastrointestinal toxicity	
Yes	93 (53.14%)
No	82 (46.86%)

pCR, pathological complete response; CR, complete response; PR, partial response; SD, stable disease; PD, progressive disease; NAC, neoadjuvant chemotherapy.

**Table 2:** Association between rs4442975 and the efficacy of NAC.

Variable	Efficacy		
	Effective (pCR + CR + PR)	Invalid (SD + PD)	OR^*a*^ (95% CI); *P*
	N (%)	N (%)	
rs4442975	140	26	
TT	101 (72.14)	20 (76.92)	1.00
GT	37 (26.43)	6 (23.08)	0.76 (0.28–2.07); 0.588
GG	2 (1.43)	0	–
Dominant			0.74 (0.27–2.01); 0.556
Additive			0.73 (0.27–1.94); 0.527

^*a*^Odds ratios (ORs) and 95% confidence intervals (CIs) were calculated using unconditional logistic regression analysis after adjusting for age, smoking, alcohol use, and menopausal status.

**Table 3:** Association between rs4442975 and bone marrow suppression by NAC.

Variable	Bone marrow suppression		
	No	Yes	OR (95% CI); *P*^*a*^
	N (%)	N (%)	
rs4442975	61	105	
TT	37 (60.66)	84 (80.00)	1.00
GT	22 (36.07)	21 (20.00)	0.39 (0.19–0.81); 0.011
GG	2 (3.28)	0	–
Dominant			0.36 (0.17–0.73); 0.005
Additive			0.38 (0.18–0.69); 0.003

^*a*^ORs and 95% CIs were calculated by unconditional logistic regression analysis after adjusting for age, smoking, alcohol use, and menopausal status.

## Data Availability

The data used to support the findings of this study are included within the article.
